# Improving Quality Indicator of Melanoma Management – Change of Melanoma Mortality-to-Incidence Rate Ratio Based on a Hungarian Nationwide Retrospective Study

**DOI:** 10.3389/fonc.2021.745550

**Published:** 2021-10-19

**Authors:** Máté Várnai, Zoltán Kiss, Rolland Gyulai, Judit Oláh, Péter Holló, Gabriella Emri, András Csejtei, István Kenessey, Angéla Benedek, Zoltán Polányi, Zsófia Nagy-Erdei, Andrea Dániel, Kata Knollmajer, György Rokszin, Ibolya Fábián, Zsófia Barcza, Csaba Polgár, Balázs Nagy, Gabriella Liszkay, Zoltán Vokó

**Affiliations:** ^1^Center for Health Technology Assessment, Semmelweis University, Budapest, Hungary; ^2^MSD Pharma Hungary Ltd., Budapest, Hungary; ^3^Department of Dermatology, Venerology and Oncodermatology, Medical School and Clinical Center, University of Pécs, Pécs, Hungary; ^4^Department of Oncotherapy, University of Szeged, Szeged, Hungary; ^5^Department of Dermatology and Allergology, University of Szeged, Szeged, Hungary; ^6^Department of Dermatology, Venereology and Dermatooncology, Semmelweis University, Budapest, Hungary; ^7^Department of Dermatology, Faculty of Medicine, University of Debrecen, Debrecen, Hungary; ^8^Department of Oncoradiology, Markusovszky University Teaching Hospital, Szombathely, Hungary; ^9^National Institute of Oncology, Budapest, Hungary; ^10^RxTarget Ltd., Szolnok, Hungary; ^11^Department of Biomathematics and Informatics, University of Veterinary Medicine, Budapest, Hungary; ^12^Syntesia Medical Communications Ltd., Budapest, Hungary; ^13^Department of Oncology, Semmelweis University, Budapest, Hungary

**Keywords:** melanoma, mortality-to-incidence ratio, mortality rate, incidence rate, Hungary

## Abstract

**Introduction:**

The incidence of melanoma has been increasing in the last decades. A retrospective Hungarian epidemiological study provided real-world data on incidence and mortality rates. There have been changing trends in incidence in Hungary in the last decade and mortality decreased, shifting mortality-to-incidence rate ratios (MIR). MIR is an indicator of cancer management quality.

**Objectives:**

Our aim is to show the changes of melanoma MIR in Hungary between 2011 and 2018 and to compare the real-world evidence-based results of our Hungarian nationwide retrospective study with other European countries.

**Methods:**

MIR is calculated from the age-specific standardized incidence and mortality rates from our study. Annual MIR values are presented for the total population and for both sexes between 2011 and 2018, along with 95% confidence intervals. Comparison with European countries are shown for 2012 and 2018 based on the GLOBOCAN database and Eurostat health care expenditure per capita data.

**Results:**

MIR decreased by 0.035 during the study years. The decrease was same in both sexes (0.031). Male had higher MIRs in all study years. In both 2012 and 2018, Hungarian MIR in both sexes was lower than the European Union average (males: 0.192 vs. 0.212 and 0.148 vs. 0.174 respectively, women: 0.107 vs. 0.129 and 0.083 vs. 0.107 respectively).

**Discussion:**

Hungarian mortality-to-incidence ratio is the lowest in Central and Eastern Europe and is close to the level of Western and Northern European countries. The results are driven by the high number of new diagnosed melanoma cases.

## Introduction

In recent decades, the incidence of melanoma has been continuously increasing (1–3). Melanoma was the 5th most common cancer for females in Europe in both 2012 and 2018 (estimated age-adjusted standardized rates [ASR] per 100,000: 13.1 and 17.7 respectively) and 9th in 2012, 7th in 2018 for males (estimated ASR: 13.1 and 18.4 respectively) (4, 5). Despite the growing trends, there are signs that incidence of melanoma is stabilizing in some regions, e.g. in North America or Scandinavia (2, 3). The mortality of melanoma showed increasing trends, similarly to incidence (6, 7), however, there are regions where mortality is decreasing (8–10), including Europe (ASR per 100,000 in 2012: 3.1 for males, 1.9 for females, in 2018: 2.8 for males, 1.7 for females) (4, 5). Our recent epidemiological study presented a change in Hungarian melanoma incidence trend in the last decade and confirmed a decrease in mortality, as well (11).

While incidence and mortality are good measures and useful tools for comparability, the rates themselves may not show the quality of care, as mortality can increase in parallel with rising incidence, despite that the ratio is not changing (12). Mortality-to-incidence ratios (MIR) have been used for more than 40 years, first as a measure of completeness of cancer registries (13, 14). Where quality of data is good, MIR can be a valid proxy indicator for survival estimates (calculated as 1-MIR) (14–16), and has already been used in multiple cancer types (17–21), including melanoma (22–24). Despite this, the use of MIR for survival estimation is mathematically questionable as the calculation does not use the same population for incidence and mortality (13).

MIR is a good tool for international comparisons, as it is easily calculable with access to good quality data and using standardized rates which allows cross-country comparison (25). Studies on various types of cancer in OECD countries also discussed the potential of MIR as a measure for evaluation the success of cancer surveillance and screening (16, 17, 26). Studies generally found reverse correlation between health-related expenditure and MIR or development and MIR in multiple cancer types (18, 20, 23). The GLOBOCAN database of the International Agency for Research on Cancer (IARC) contains the best available evidence on age-standardized cancer-specific incidence and mortality. There have been analyses using the GLOBOCAN data to compare the MIRs of participating countries (4, 23, 27). The latest versions of studies about European countries uses the 2012 and 2018 GLOBOCAN data (4, 5) which could serve as a base for MIR analysis and evaluation of changes between 2012 and 2018.

Our aim is to use Hungarian real-world data from our recent “Real-world retrospective Analysis on the Melanoma Management pattern on the basis of countrywide Hungarian registry (RAMM)” study that is based on the National Health Insurance Fund (NHIF) database and that of the Central Statistical Office (CSO) from 2011 to 2018 instead of calculating from estimates. Using population level data allows us to measure the size and change of MIR and position Hungary accurately among European countries in aspect of MIR. Previously we found that age-standardized incidence rates were higher in males and varied between 28.28 and 34.57/100,000 person-years (PYs), and between 22.63 and 26.72/100,000 PYs in females and mortality rates varied between 4.73 and 5.98/100,000 PYs in males and between 2.33 and 3.20/100,000 PYs in females (11).

## Materials and Methods

The RAMM study was a nationwide, retrospective, longitudinal study conducted using the databases of the Hungarian NHIF and CSO. The NHIF database covers the entirety of the Hungarian population with social insurance coverage with ICD-10 coding information about in- and outpatient visits. The CSO database contains annual cause-specific mortality data of Hungarian citizens.

Our epidemiological study included patients with melanoma (ICD-10 code: C43) diagnosed between January 1, 2011 and December 31, 2018 who were at least 20 years old at the time of diagnosis. A minimum of two occurrences of ICD-10 C34 in medical records were required for inclusion. Hungarian population sizes for standardization calculations and data on cause-specific mortality (date, crude numbers) were obtained from the CSO. Calculated incidence and mortality rates from crude numbers were expressed as age-standardized rates (ASRs, per 100,000 PYs). ASRs are presented using two different population sets: the European Standard Population from (ESP) 1976 and 2013. Different standardization was necessary to allow for a direct comparison with recent GLOBOCAN data that uses the ESP 1976 dataset for European analyses and to evaluate the change of melanoma MIR in recent years (ESP 2013). More details on the identification of patients with malignant melanoma were described in our previous article where we reported the main epidemiology results of our study (11).

Mortality-to-incidence ratio was calculated by dividing the age-standardized mortality rate by the age-standardized incidence rate per 100,000 PYs. Annual MIR values are presented for the total population and for both sexes between 2011 and 2018. Results from 2012 and 2018 are compared with European countries using GLOBOCAN data from the same years. European regional country division is based on the GLOBOCAN publications. We also investigate the correlation between health expenditure per capita and MIR for European countries in 2012 and 2018, using the Eurostat database for expenditure data. Countries not covered in the Eurostat database will be excluded from the analysis (28).

### Statistical Analysis

Linear regression was applied to estimate the annual change of MIR: the outcome variable was the MIR, the explanatory variable was the observed year. Statistical significance level was set at the probability of 0.05. We used R version 4.0.2 (2020-06-22) for calculations. The correlation between health expenditure per capita and MIR is calculated with the Spearman’s rank correlation method.

## Results

### Annual Hungarian Mortality-to-Incidence Ratios

**Figure 1:**
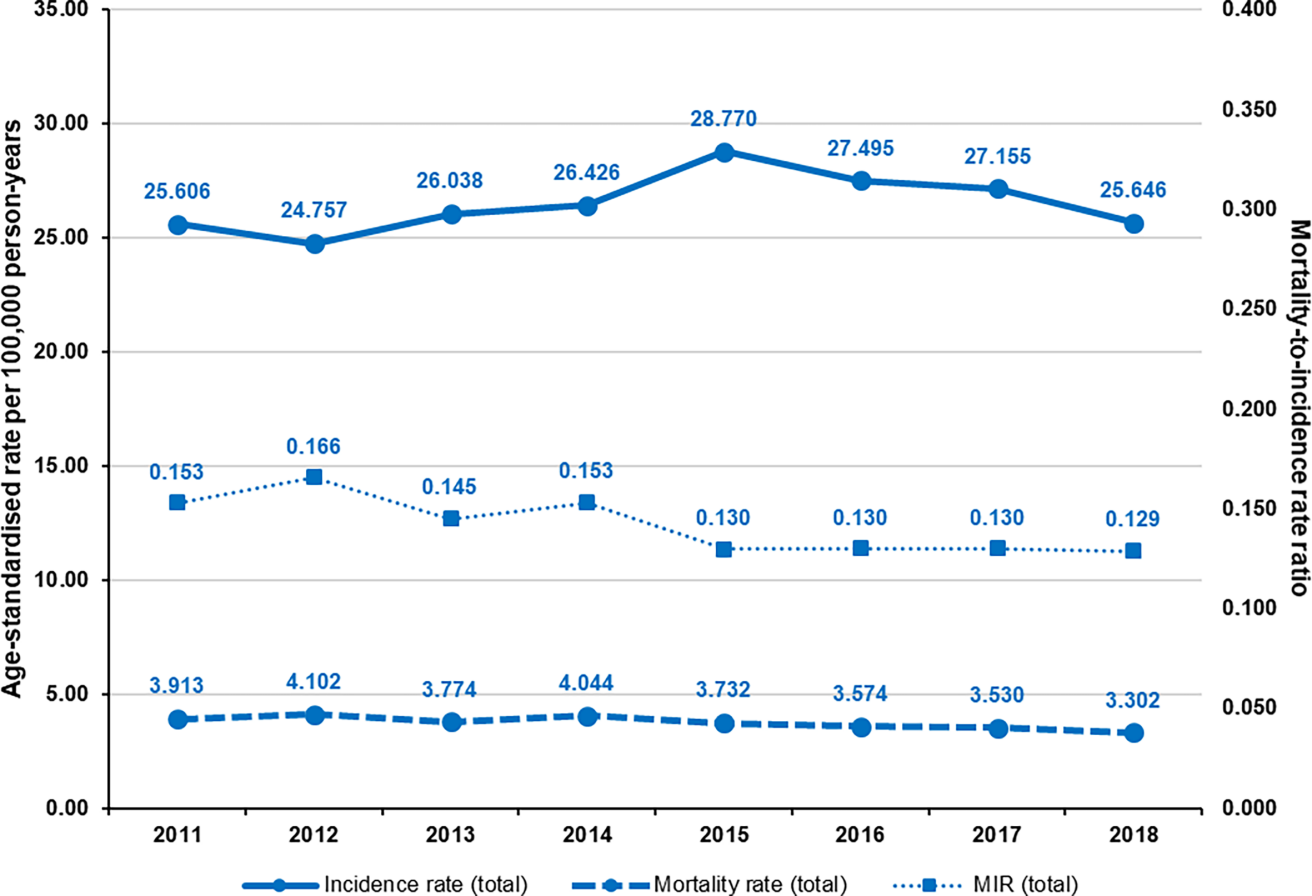
Age-standardized incidence rates, mortality rates, and mortality-to-incidence ratios of melanoma in Hungary between 2011 and 2018 (per 100,000 person-years, using ESP 2013).

Melanoma incidence was increasing until 2015, which was followed by a gradual decrease until the end of the study period (ESP 2013), while melanoma mortality started to decrease in 2014. MIR varied between 0.145 and 0.166 in the total melanoma population from 2011 to 2014 and remained constant at around 0.130 since 2015 (**Figure 1**). The regression coefficient was -0.005, corresponding to a change of -0.035 for the whole study period (95% CI: -0.056 to -0.013; p=0.0076).

**Figure 2:**
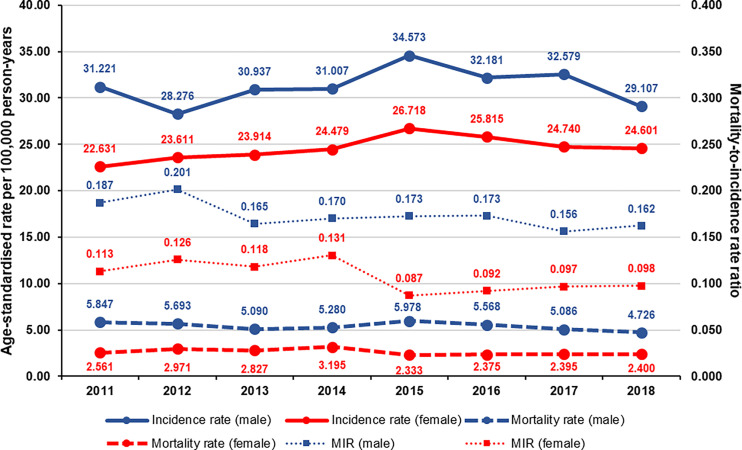
Age-standardized incidence rates, mortality rates, and mortality-to-incidence ratios of melanoma per sex in Hungary between 2011 and 2018 (per 100,000 person-years, using ESP 2013).

MIR was lower in women than in men in all study years, with MIR values ranging from 0.156 to 0.201 in men and from 0.087 to 0.131 in women (**Figure 2**). Sex-specific MIR trends had minor differences from the total population. MIR in male patients had a peak in 2012, which was followed by a larger drop, and a small and steady increase until 2016. MIR among female patients was increasing between 2011 and 2014, after which it considerably decreased to 0.087 by 2015 and slowly increased until the end of the study period. The change of MIR was equal for males (-0.031; 95% CI: -0.059 to -0.003; p=0.0335) and for females (-0.031; 95% CI: -0.066 to 0.003; p=0.0686).

MIR values based on ESP 1976 standardization showed similar trends, with MIRs of 0.150 (male 0.194, female 0.107) in 2012, and 0.113 (male 0.148, female 0.082) in 2018 (**Supplementary Table 1**).

### Hungarian MIR Compared to European Countries

**Figure 3:**
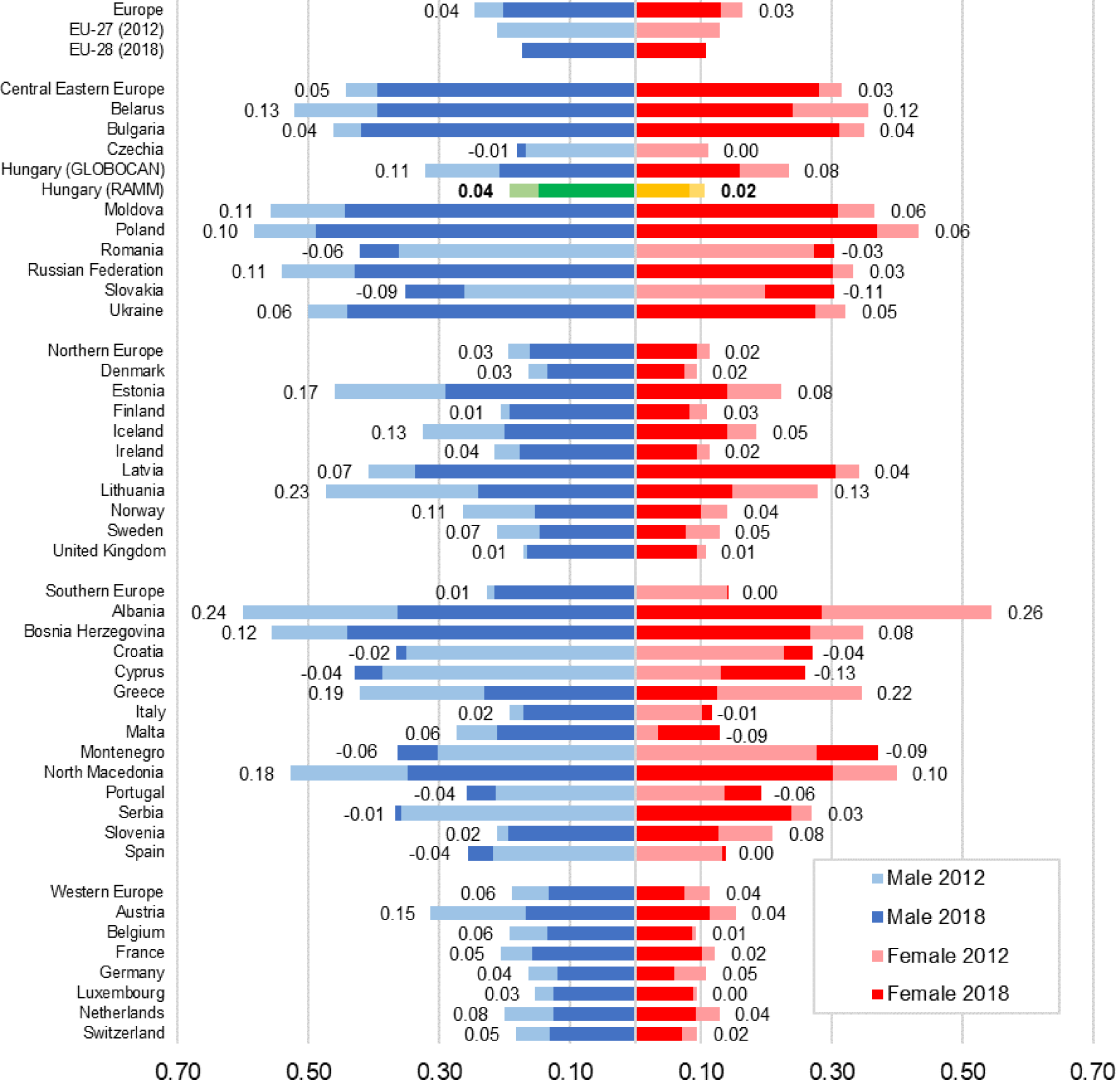
Mortality-to-incidence ratios of melanoma by sex in Europe in 2012 and 2018 using the ESP 1976 dataset, all countries with data (numbers indicate the difference between 2018 and 2012 values). Note: Hungary (GLOBOCAN) refers to the publication of Ferlay et al. (4); Hungary RAMM refers to our findings. Our findings are highlighted with different coloration for distinction: males are green (2012 light, 2018 dark), women are yellow (2012 light, 2018 dark).

The age-specific burden of melanoma is similar in European countries (**Supplementary Figure 1**) (29), with mortality rates being more similar to each other than incidence. The average European MIR in 2012 was 0.245 for males and 0.163 for females and changed to 0.202 for males and 0.130 for females in 2018. Western Europe (WE) and Northern Europe (NE) had the lowest MIRs both in 2012 and 2018. Central and Eastern Europe (CEE) had the highest MIRs in both years. In 2018, Hungary had the lowest MIR in the CEE region for both sexes), which was comparable to the WE and NE average (males 0.162 and 0.133, females 0.095 and 0.076 in 2012 and 2018; respectively). In 2018, MIR in Hungary ranked 8th among European countries for males, and 6th for females (**Figure 3** and **Table 1**). With the exception of female patients in Southern Europe, MIR decreased in all regions between 2012 and 2018. The change in MIR was comparable to the aggregated European values, indicating a steady improvement in the quality of melanoma management on the continent (**Table 1**).

**Table 1:** Mortality-to-incidence ratios of melanoma by sex in European regions in 2012 and 2018 using the European Standard Population 1976 dataset (4, 5).

Region	Male			Female		
	2012	2018	Difference	2012	2018	Difference
**Hungary (RAMM)**	0.194	0.148	0.046	0.107	0.082	0.025
**Europe**	0.246	0.203	0.043	0.164	0.130	0.033
**EU-27 (2012)/EU-28 (2018)**	0.212	0.174	0.038	0.130	0.107	n. a.
**Western Europe**	0.188	0.133	0.056	0.114	0.076	0.038
**Northern Europe**	0.194	0.162	0.031	0.113	0.095	0.019
**Central and Eastern Europe**	0.443	0.395	0.048	0.317	0.282	0.035
**Southern Europe**	0.228	0.216	0.012	0.140	0.143	-0.003

n.a., not appicable.

**Figure 4:**
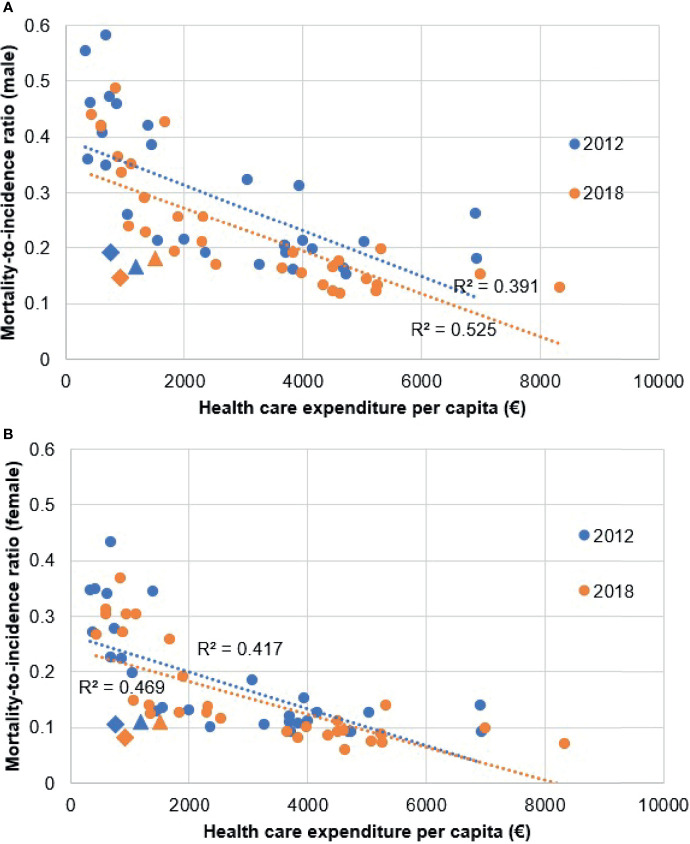
Correlation between melanoma mortality-to-incidence ratios and health expenditure per capita in 2012 and 2018 using the ESP 1976 dataset. **(A)** male patients, **(B)** female patients. Diamond shaped plot points indicate Hungary, triangle shaped plot points indicate Czechia.

The results of the Spearman’s rank correlation calculation show strong negative correlation between health expenditure per capita and MIR in both 2012 (males R=-0.663, p<0.000, females R=-0.699, p<0.000) and 2018 (males R=-0.800, p<0.000, females R=-0.761, p<0.000) regardless of sex (**Figure 4**). Our results are in line with the findings of Forsea et al. (23). Health expenditure (HE) per capita increased in all countries in the period with the exception of Greece. There were no HE data on Malta and Slovenia for 2012, thus those plot points are only visualized for 2018. R^2^ in 2012 was 0.391 in males, 0.417 in females and in 2018 0.525 in males, 0.470 in females. Hungary and Czechia showed as notable exceptions in both 2012 and 2018, with noticeably lower HE per capita from other countries with comparable MIR. The difference from other countries was more distinguishable in males than in females.

## Discussion

Our nationwide study was the first to examine the incidence and mortality of melanoma as well as the MIR and its changes over a 8-year period in Hungary (23). We found a decrease of 0.035 in MIR during the whole study period with similar decreases in both sexes and male patients having higher MIRs in all study years. Hungarian MIR was lower than the European average both in 2012 and 2018 in both sexes.

The mortality-to-incidence ratio provides an alternative means to assess the burden of a disease and quality of care by presenting mortality after accounting for incidence. MIR can be interpreted as a quality indicator used for international comparisons and for the identification of racial and sex disparities in cancer outcomes (12, 14–16). Low values can be attributed to the success of screening, quality of care, or access to modern melanoma therapies. In their analysis, Forsea et al. calculated MIRs for melanoma across Europe based on 2008 GLOBOCAN data and found the highest ratios in Central and Eastern Europe (0.35 on average) and the lowest ratios in Western Europe (0.13 on average). Furthermore, they found a strong inverse correlation between MIRs and total health expenditure per capita in European countries (r=–0.76, p<0.05) (23). Good quality data is required for internationally comparable results (25). Our study showed that using real-world population data can result in a somewhat different measure than using an estimate (**Figure 3**), as the incidence and mortality rates in the RAMM study were lower than the GLOBOCAN estimates. In the current study, MIRs varied between 0.156 and 0.201 in men and between 0.087 and 0.131 in women during the 2011–2018 study period, showing a decreasing trend in both sexes. These values are comparable to MIRs reported by Forsea et al. for Western European countries, and far below the data reported for CEE countries. Furthermore, while the volume and accuracy of melanoma diagnosis in Hungary resulted in higher absolute incidence rates compared to other CEE countries, our study shows a decline in annual incidence rates starting from 2015. Our findings show very similar cases to the numbers of the National Cancer Registry (e.g. 2012: Registry 2291 vs 2360, in 2018: Registry 2778 vs 2528) which gathers epidemiological data in Hungary for over 20 years (30). The simultaneous decrease in MIR suggests that apart from incidence, melanoma mortality also started to decrease recently, which may be attributed to several factors including the relatively high access to innovative therapies and high health expenditure per capita compared to neighboring CEE countries as reported by Kandolf Sekulovic et al. (31) or possibly the success of secondary prevention in screening and diagnosis.

An important factor shaping Hungarian incidence patterns could be the successful local implementation of the Euromelanoma educational and prevention campaign, which has been active for more than two decades across Europe. Hungary joined the Euromelanoma campaign relatively early in 2007, which led to the subsequent increase in the number of screening and awareness campaigns. The first Euromelanoma campaign in Hungary was organized in 2009 (see **Supplementary Table 2**) (32), and the campaign has been considered highly effective in increasing the timely diagnosis of clinically suspected melanoma ever since (33). The Hungarian Dermatological Society performed over 30,000 birthmark screenings free of charge with the involvement of more than 100 volunteering dermatologists since 2007 (34). Early detection has an important effect on pathological prognostic factors including melanoma (Breslow) thickness and Clark level, both of which are strongly associated with survival (35). The Hungarian National Institute of Oncology analyzed cutaneous malignant melanoma cases and found a significant decrease in mean Breslow thickness from 2.2 mm to 1.6 mm (p<0.001) and a non-significant decrease in Clark level between 1998 and 2008 (36). Accordingly, the earlier detection of the disease led to an increase in incidence and a decrease in mortality, as shown by our previous study (11). The improvement in melanoma management was also reflected by the increase in the mean age of the total prevalent melanoma population, suggesting that patients die of other causes, which results in an increasing gap between all-cause and cause-specific mortality. Disability-adjusted life-year (DALY) analyses have found that the burden of melanoma affects male patients and elder patients the greatest. The studies call for more prevention efforts to diagnose melanoma at earlier stages, as life-years spent with disability is lower than life-years lost. Earlier diagnoses could lead to reduced melanoma burden, with life-years lost turning into life-years with disability (37–39). The studies were published before the onset of novel immunotherapeutic agents, when researches were ongoing in both drug therapies and predictive markers that may also affect melanoma DALY (39).

Apart from early detection, the availability of modern pharmaceutical therapies has also had a significant effect on melanoma survival, especially in the metastatic stage. Targeted therapies (BRAF and MEK-inhibitors) and immune checkpoint inhibitors (ipilimumab, nivolumab, pembrolizumab) have provided significant survival benefits for patients with stage III–IV melanoma (40). Access to modern treatments is an indicator of the performance of healthcare systems (31). The IMS-IQVIA database shows that targeted therapies and immune checkpoint inhibitors were introduced into Hungarian clinical practice shortly after their introduction in Western European countries, which is reflected by similar MIR values in these populations (41). Furthermore, the increasing complexity of advanced cancer care brought about several new challenges and revealed disparities in everyday clinical practice. Therefore, in line with international initiatives (42), the European Cancer Organization essential requirements for quality cancer care expert group recommended that advanced melanoma management be only carried out in, or in collaboration with, specialized melanoma centers where both a core multidisciplinary team and an extended team of allied professionals were available, and which were subject to quality and audit procedures (43). In the interest of high-quality comprehensive cancer control, Hungarian healthcare organizations implemented the recommendations for high-quality patient care in melanoma management according to progressivity level (44) and nominated 7 clinics as “Melanoma Centres” to promote clinical experience with modern immune and targeted therapies (45). Improved melanoma management as shown by our results reflects the validity and success of centre-based melanoma care (11). Furthermore, almost the entirety of the Hungarian population is covered by social insurance with full access to reimbursed therapies. Individuals in risk groups can get screening without age limitations.

Our study found higher MIR values among male patients during the whole study period, which is in line with observations from other countries. Gender-specific differences in mortality have been reported for the majority of tumour types (46). Data from the American Academy of Dermatological National Skin Cancer Screening Program showed that although men aged 50 years or older constituted only a quarter of melanoma screenings, they accounted for almost half of confirmed diagnoses (47). Furthermore, Rat et al. reported significantly lower adherence to targeted skin cancer screening programs compared to women (48), and the majority of attendants at Euromelanoma screening campaigns were also found to be female in 2009–2010 (73% in Hungary) (33). Skin cancer risk awareness, the involvement of primary healthcare physicians and dermatologists, and comprehensive information and education provided by the media are important elements in melanoma diagnosis.

**Figure 5:**
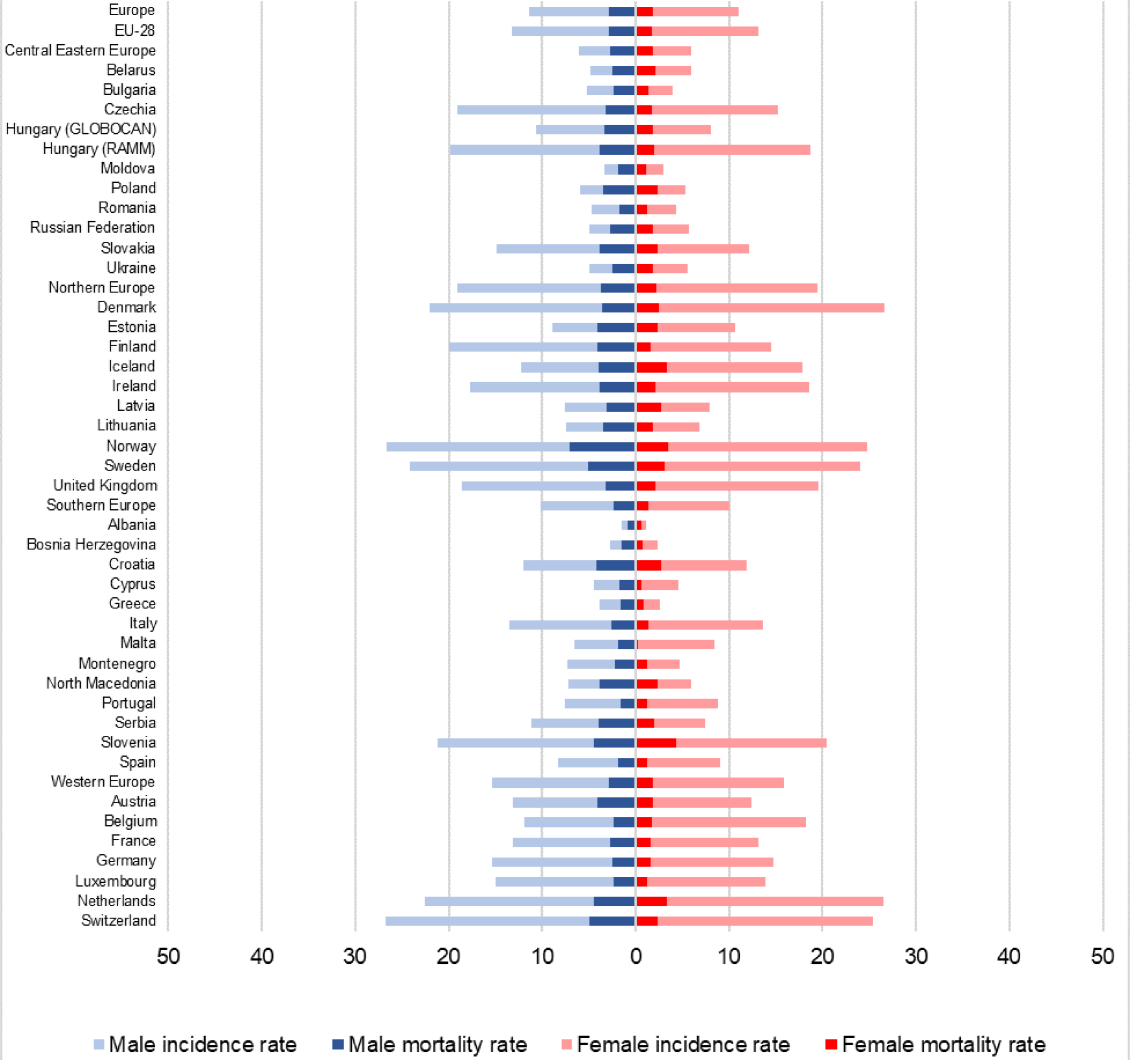
Age-standardized melanoma incidence and mortality rates by sex in Europe, 2018 (ESP 1976 dataset).

Hungary and the Czech Republic were the only two CEE countries with comparable MIR to WE and NE countries in both sexes (**Figure 3**). **Figure 5** shows that these two countries had the highest incidence rates in the CEE region, which may be attributed to successful screening campaigns. Access to innovative melanoma medicines (targeted therapies and/or immunotherapies) is ≥90% in both countries (percentages show patients treated with said drugs), thus diagnosed patients have access to the best available therapies that shows in mortality rates (31, 49). While there are studies, which found that total health expenditure or expenditure on oncology drugs correlates with MIR, none is concerning melanoma-specific treatments (23, 50). Our results using Eurostat HE data also showed that although higher total HE per capita correlated with better MIR values, in line with the findings of Forsea et al. (23). MIR in Hungary and the Czech Republic was substantially better than it would have been expected based on their HE per capita (**Figure 4**). As the age-specific incidence rates in 2012 showed, was comparable to several other European countries, e.g. to Germany and the UK (**Supplementary Figure 1**). This suggests successful screening practices, possibly due to the Euromelanoma campaigns (33). However, apart from these exceptions, evidence suggests that higher volume of expenditure on melanoma drugs leads to better outcomes.

MIR as a tool has its limitations. Despite previous proposals, the MIR should not be used in survival estimations as the population included in incidence and mortality are not necessarily the same, as advised by Ellis and colleagues (13). We would rather recommend using MIR as a rapid tool for trend evaluations and international comparisons for melanoma management, due to the availability of epidemiological data.

In conclusion, the Hungarian mortality-to-incidence ratio of melanoma was the lowest in Central and Eastern Europe and comparable to that of Western and Northern European countries. The results are potentially due the outstanding number of new diagnosed melanoma cases as a success of melanoma screening campaigns in Hungary. Our results show that efforts for early detection of melanoma can improve mortality-to-incidence rates. Further analyses could explore the effect of the access to health care resources in the therapeutic area.

## Data Availability Statement

The raw data supporting the conclusions of this article will be made available by the authors, without undue reservation.

## Author Contributions

MV: Conceptualization, methodology, writing—original draft. ZV, GL, ZK, and CP: Supervision, writing. RG, JO, PH, GE, and AC: Conceptualization, validation. IK: Data validation. AB, ZP, ZN-E, and AD: Conceptualization, validation of data. ZV and BN: Methodology, supervision. GR and IF: Data curation. ZB: Writing—review and editing. KK: Managing manuscript. MV and KK: Proofing. All authors contributed to the article and approved the submitted version.

## Funding

The authors declare that this study received funding from MSD Pharma Hungary.

## Conflict of Interest

MV, ZK, ZP, AD, AB, KK, and ZN-E were employed by the company MSD Pharma Hungary. GR and IF were employed by the company RxTarget Ltd. ZB was employed by the company Syntesia Medical Communications Ltd.

The remaining authors declare that the research was conducted in the absence of any commercial or financial relationships that could be construed as a potential conflict of interest.

The authors declare that this study received funding from MSD Pharma Hungary. The funder had the following involvement with the study: in study design, data collection and analysis, decision to publish, or preparation of the manuscript.

## Publisher’s Note

All claims expressed in this article are solely those of the authors and do not necessarily represent those of their affiliated organizations, or those of the publisher, the editors and the reviewers. Any product that may be evaluated in this article, or claim that may be made by its manufacturer, is not guaranteed or endorsed by the publisher.
