# Supplementary material for: Improving Quality Indicator of Melanoma Management – Change of Melanoma Mortality-to-Incidence Rate Ratio Based on a Hungarian Nationwide Retrospective Study
Source: Front Oncol. 2021 Oct 19;11:745550. doi: 10.3389/fonc.2021.745550 (PMC8570304; doi:10.3389/fonc.2021.745550)
Supplement: Supplementary file 1 [file DataSheet_1.docx]

Supplementary Material

Supplementary Figure 1: Age-specific incidence and mortality rates per 100,000 in Hungary and a few European countries* in 2012. A: incidence rate, B: mortality rate

*
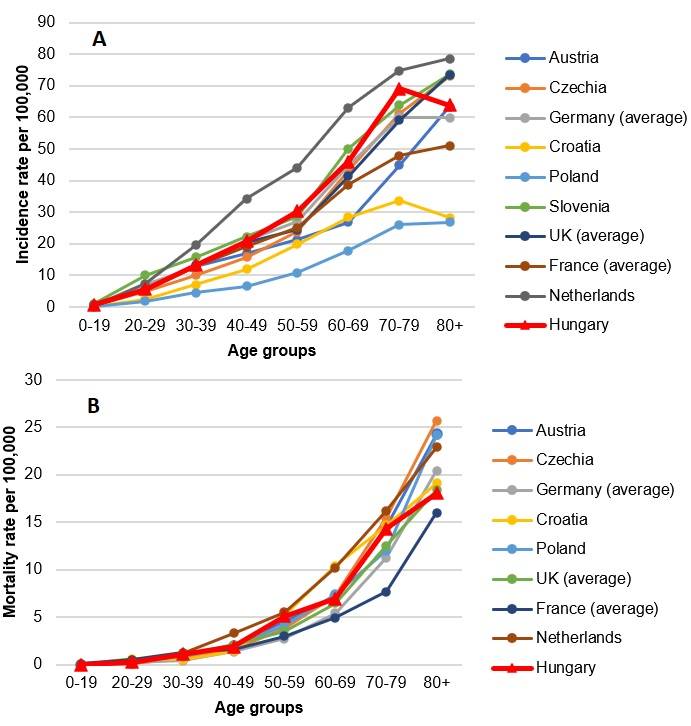
*

*Germany average: Bavaria, Berlin, Bremen, Hamburg, Hessen, Lower Saxony, North Rhine-Westphalia, Rhineland-Palatinate, Saarland, Schleswig-Holstein; UK average: England, Northern Ireland, Scotland; France average: Average of Bas-Rhin, Belfort, Calvados, Doubs, Gironde, Haut-Rhin, Herault, Isere, Lille Area, Limousin, Loire-Atlantique/Vendée, Manche, Poitou-Charentes, Somme, Tarn; Hungary results from the RAMM study
