# Supplementary material for: Improving Quality Indicator of Melanoma Management – Change of Melanoma Mortality-to-Incidence Rate Ratio Based on a Hungarian Nationwide Retrospective Study
Source: Front Oncol. 2021 Oct 19;11:745550. doi: 10.3389/fonc.2021.745550 (PMC8570304; doi:10.3389/fonc.2021.745550)
Supplement: Supplementary file 2 [file Table_1.docx]

Supplementary Material

Supplementary Table 1: Mortality-to-incidence ratios between 2011 and 2018 and total change in the period in the total population and per sex, populations based on the ESP 1976

| **Population** | **Year** | | | | | | | | **2011-2018** |
| --- | --- | --- | --- | --- | --- | --- | --- | --- | --- |
|  | **2011** | **2012** | **2013** | **2014** | **2015** | **2016** | **2017** | **2018** | **Total change (95% CI; p)** |
| **Total** | 0.136 | 0.150 | 0.128 | 0.134 | 0.113 | 0.112 | 0.111 | 0.113 | -0.035  (-0.057, -0.013; 0.0082) |
| **Male** | 0.173 | 0.194 | 0.155 | 0.155 | 0.159 | 0.151 | 0.141 | 0.148 | -0.038  (-0.068, -0.008; 0.0215) |
| **Female** | 0.095 | 0.107 | 0.098 | 0.110 | 0.071 | 0.078 | 0.076 | 0.082 | -0.029  (-0.060, -0.002; 0.0618) |
