# Supplementary material for: Improving Quality Indicator of Melanoma Management – Change of Melanoma Mortality-to-Incidence Rate Ratio Based on a Hungarian Nationwide Retrospective Study
Source: Front Oncol. 2021 Oct 19;11:745550. doi: 10.3389/fonc.2021.745550 (PMC8570304; doi:10.3389/fonc.2021.745550)
Supplement: Supplementary file 3 [file Table_2.docx]

Supplementary Material

Supplementary Table 2: First year of Euromelanoma campaign and MIR in 2018 in Central and Eastern European countries (4, 29)

| **Country** | **1st year of Euromelanoma campaign** | **MIR in 2018 (rank in CEE)** | |
| --- | --- | --- | --- |
|  |  | **Male** | **Female** |
| Czechia | 2001 | 0.181 (2) | 0.111 (2) |
| Slovakia | 2004 | 0.353 (3) | 0.305 (5) |
| Bulgaria | 2006 | 0.419 (4) | 0.313 (7) |
| Poland | 2006 | 0.488 (8) | 0.369 (8) |
| Russia | 2007 | 0.429 (5) | 0.303 (4) |
| Hungary | 2009 | 0.148 (1) | 0.082 (1) |
| Ukraine | 2009 | 0.442 (6) | 0.275 (3) |
| Moldova | 2010 | 0.444 (7) | 0.309 (6) |
